# A Hidden Triple Threat: A Case of Concurrent Renal Artery Stenosis, Dissection, and Pseudoaneurysm

**DOI:** 10.7759/cureus.115921

**Published:** 2026-09-07

**Authors:** Omkar S Wadekar, Sreedevi T, Gyana Ranjan Pradhan

**Affiliations:** 1 Internal Medicine, Hindustan Aeronautics Limited (HAL) Hospital, Bengaluru, IND

**Keywords:** acute kidney injury (aki), percutaneous transluminal renal angioplasty (ptra), renal artery dissection, renal artery doppler, renal artery pseudoaneurysm, renal artery stenosis, secondary hypertension

## Abstract

The simultaneous occurrence of renal artery stenosis, pseudoaneurysm, and dissection in a single native vessel is rare and poses a severe threat to renal survival. Herein, we present a case of a 50-year-old male patient on alternative medications for hypertension who presented with a three-day history of vomiting, dehydration, and 12 hours of anuria. Initially managed for dehydration and acute kidney injury, his persistent hypertension and worsening renal function prompted further investigation. Initial renal artery Doppler did not show acute changes; the patient’s clinical status necessitated hemodialysis. A re-evaluation, prompted by undisclosed family history and a neglected history of a small left kidney since childhood, led to a repeat Doppler and subsequent CT renal angiogram. Findings revealed right renal artery stenosis, dissection, and a pseudoaneurysm. Urgent percutaneous transluminal renal angioplasty (PTRA) successfully restored perfusion, resolving the patient’s renal failure and dialysis dependency. This case underscores that renal artery Doppler is highly operator-dependent and can miss critical pathologies. It highlights the necessity of maintaining a high index of suspicion for renovascular causes in hypertensive patients and emphasizes that when clinical findings are incongruent with initial imaging, further invasive diagnostic imaging is essential to ensure timely intervention.

## Introduction

In clinical practice, hypertension is one of the most common comorbidities a physician comes across. While essential hypertension is most common, approximately 10% of cases are secondary, with renovascular etiology being a prominent cause [1]. Renal artery vasculopathies comprise a heterogeneous group of uncommon but clinically significant disorders that include renal artery stenosis, pseudoaneurysm, and dissection [2]. Despite advancements in medical imaging and interventional techniques, diagnostic delays persist, often compounded by patient reliance on alternative medicines and incomplete medical histories. Early recognition of renovascular hypertension is clinically important as management ranges from medical therapy and surveillance to interventional procedures such as angioplasty, stent placement, or surgery depending on symptoms, renal perfusion, hemorrhagic risk, and lesion complexity [3].

Renal artery stenosis restricts perfusion, leading to ischemia-induced secondary hypertension, while dissections and pseudoaneurysms pose immediate threats of thrombosis, rupture, and abrupt loss of renal function. The coexistence of all three lesions in a single native vessel is exceptionally rare and severely compromises renal survival. Diagnosing such complex pathology is challenging, as initial non-invasive modalities like Doppler ultrasound may fail to capture distal or structurally complex vascular anomalies, necessitating a low threshold for advanced cross-sectional imaging.

This case underscores the critical necessity of meticulous history-taking and maintaining a high index of suspicion for renovascular pathology in patients with refractory or secondary hypertension.

## Case presentation

A 50-year-old man presented with a three-day history of vomiting, nausea, and 12 hours of anuria. Upon admission, he was clinically dehydrated and tachycardic, with high blood pressure readings. Laboratory investigations revealed acute kidney injury (AKI) with blood urea at 85 mg/dL (reference 15-39 mg/dL) and serum creatinine at 7.5 mg/dL (reference 0.5-1.3 mg/dL). A provisional diagnosis of prerenal AKI was made. Initial imaging, including an abdominal ultrasound and CT of the abdomen and pelvis (Figure 1), confirmed a shrunken left kidney, a finding the patient noted had existed since childhood without prior evaluation, and compensatory hypertrophy of the right kidney. Initial renal artery Doppler showed no stenosis (Figure 2).

**Figure 1:**
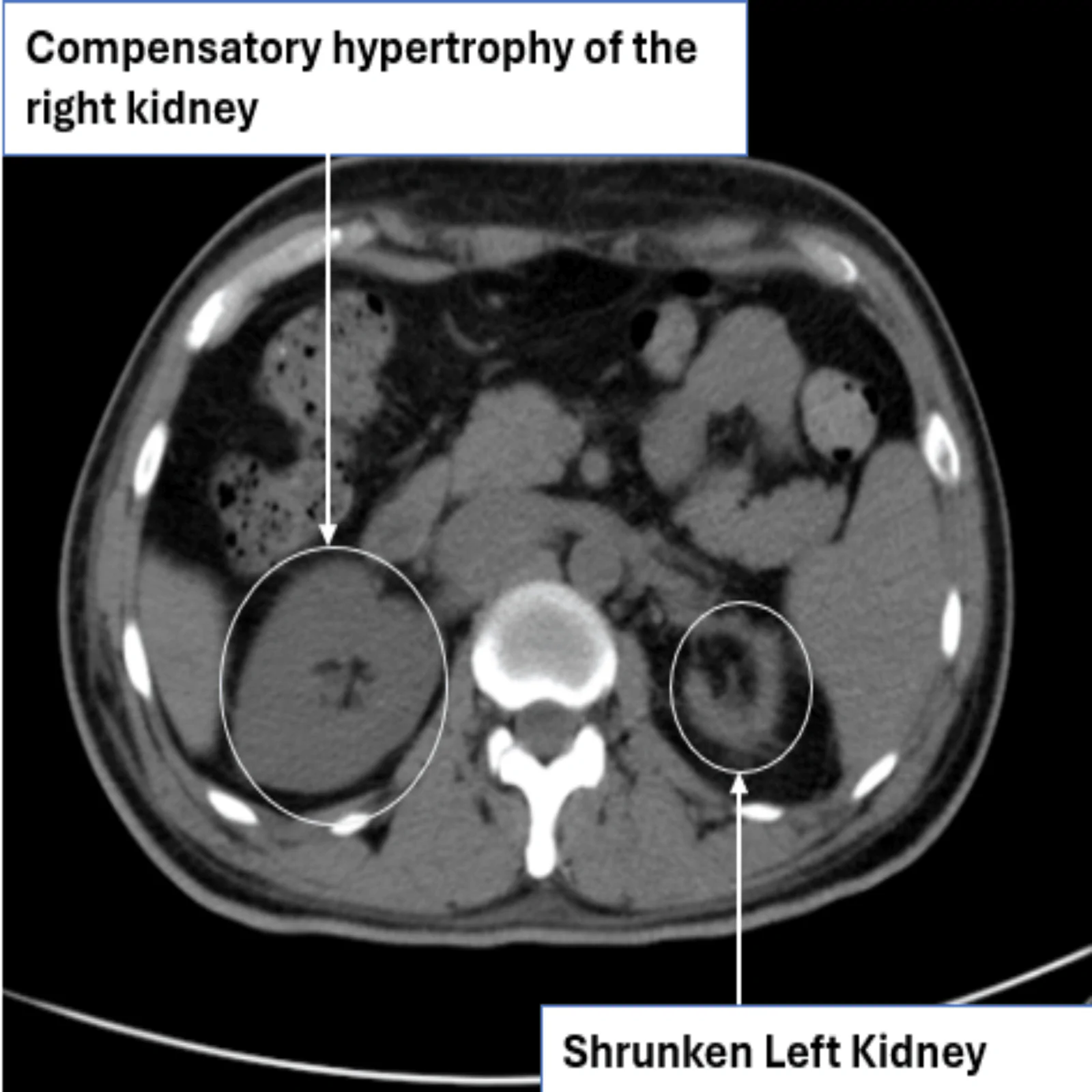
CT of the abdomen and pelvis

**Figure 2:**
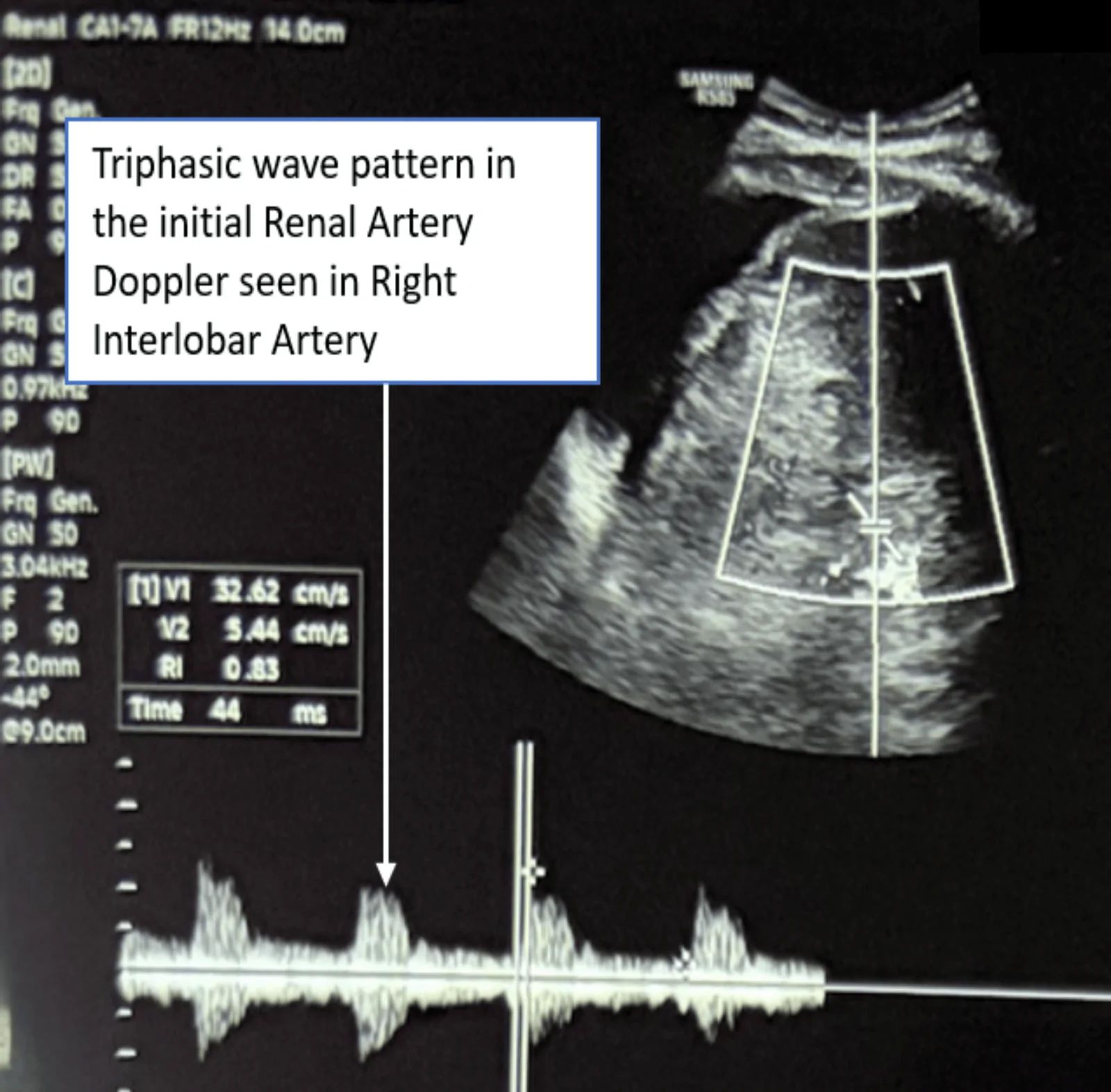
Initial right renal artery Doppler showing a normal triphasic high-resistance waveform in the interlobar artery, which falsely suggested an unimpaired proximal arterial flow.

Despite aggressive fluid resuscitation and supportive care, the patient remained anuric with progressive metabolic acidosis, necessitating hemodialysis. The patient was discharged on thrice-weekly hemodialysis.

Upon follow-up, probing multiple times and exhaustive history-taking revealed a significant family history of paternal early-onset renal disease (dialysis-dependent by late 40s) and long-term use of alternative medications (homeopathic medicine, Rauwolfia serpentina) for hypertension. Given the patient’s history of a solitary functioning kidney, chronic hypertension, and new-onset anuria, a renovascular cause was strongly suspected. A repeat renal artery Doppler suggested a parvus-tardus waveform in the right renal artery (Figure 3). A subsequent CT renal angiogram (Figure 4) provided precise anatomical delineation of a complex 'triple threat': right renal artery stenosis, dissection, and a pseudoaneurysm. The imaging demonstrated that the right main renal artery was normal for approximately 15 mm from its origin. Distal to this point, the vessel widened for a length of 42 mm up to its trifurcation, revealing an enlarged hypodense false lumen and a severely narrowed true lumen. This resulted in a 70-80% luminal narrowing over a span of 35 mm. Additionally, a focal rounded area of contrast opacification anterior to the dissecting flap was noted, representing partial opacification of the otherwise thrombosed false lumen. The scan also revealed a possible extension of the dissection into the superior segmental branch of the right renal artery, causing an additional 50% luminal narrowing at its origin.

**Figure 3:**
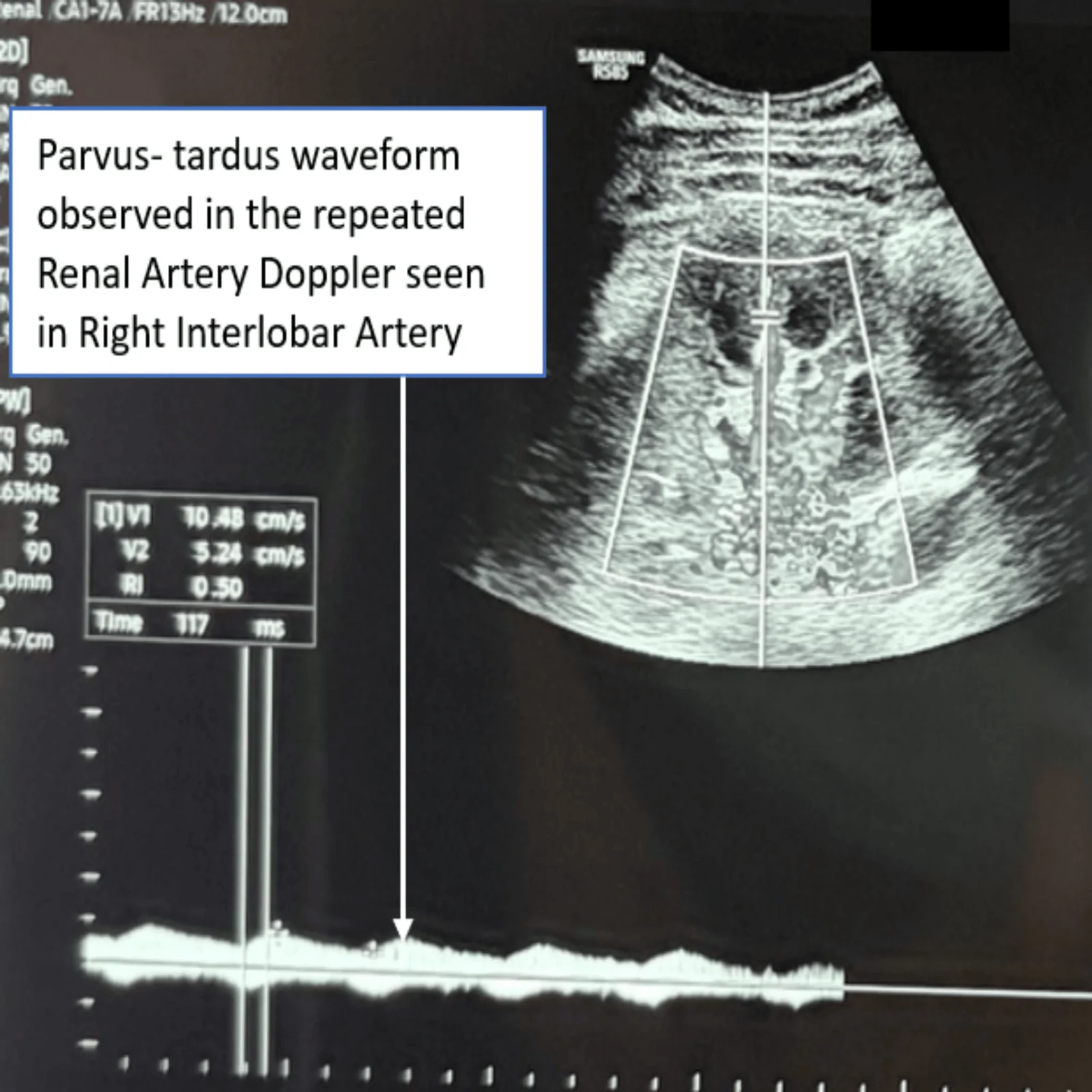
Repeat right renal artery Doppler demonstrating a parvus-tardus waveform. This dampened, low-amplitude, and delayed systolic peak reflects a hemodynamically significant stenosis proximal to the sampling site, restricting adequate perfusion.

**Figure 4:**
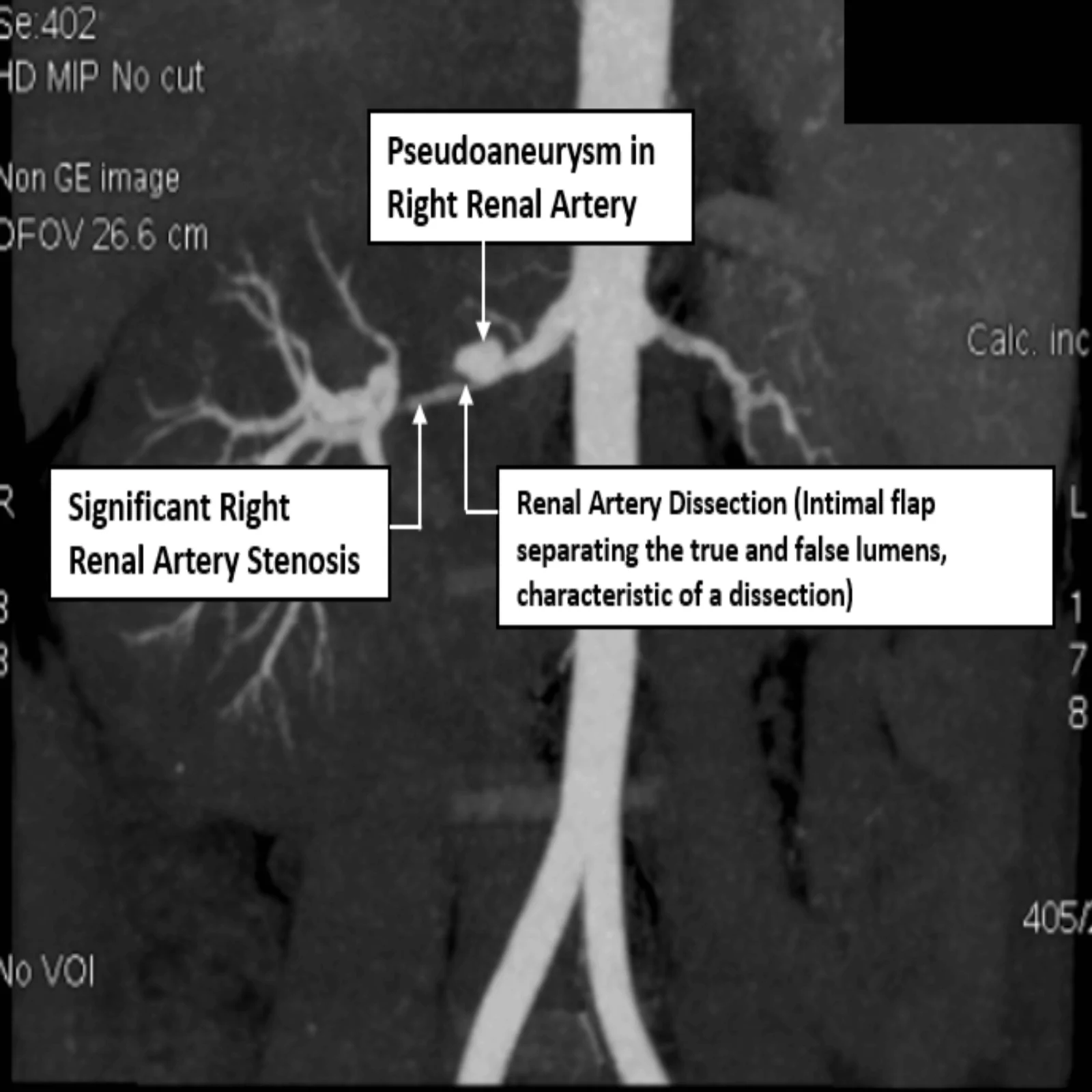
CT renal angiogram

The patient was referred for intervention. A diagnostic renal angiogram confirmed the complex pathology. The right renal artery was successfully cannulated and treated via percutaneous transluminal renal angioplasty (PTRA), utilizing two drug-eluting stents. The procedure achieved successful revascularization with no residual stenosis and a clear nephrogram (Figure 5).

**Figure 5:**
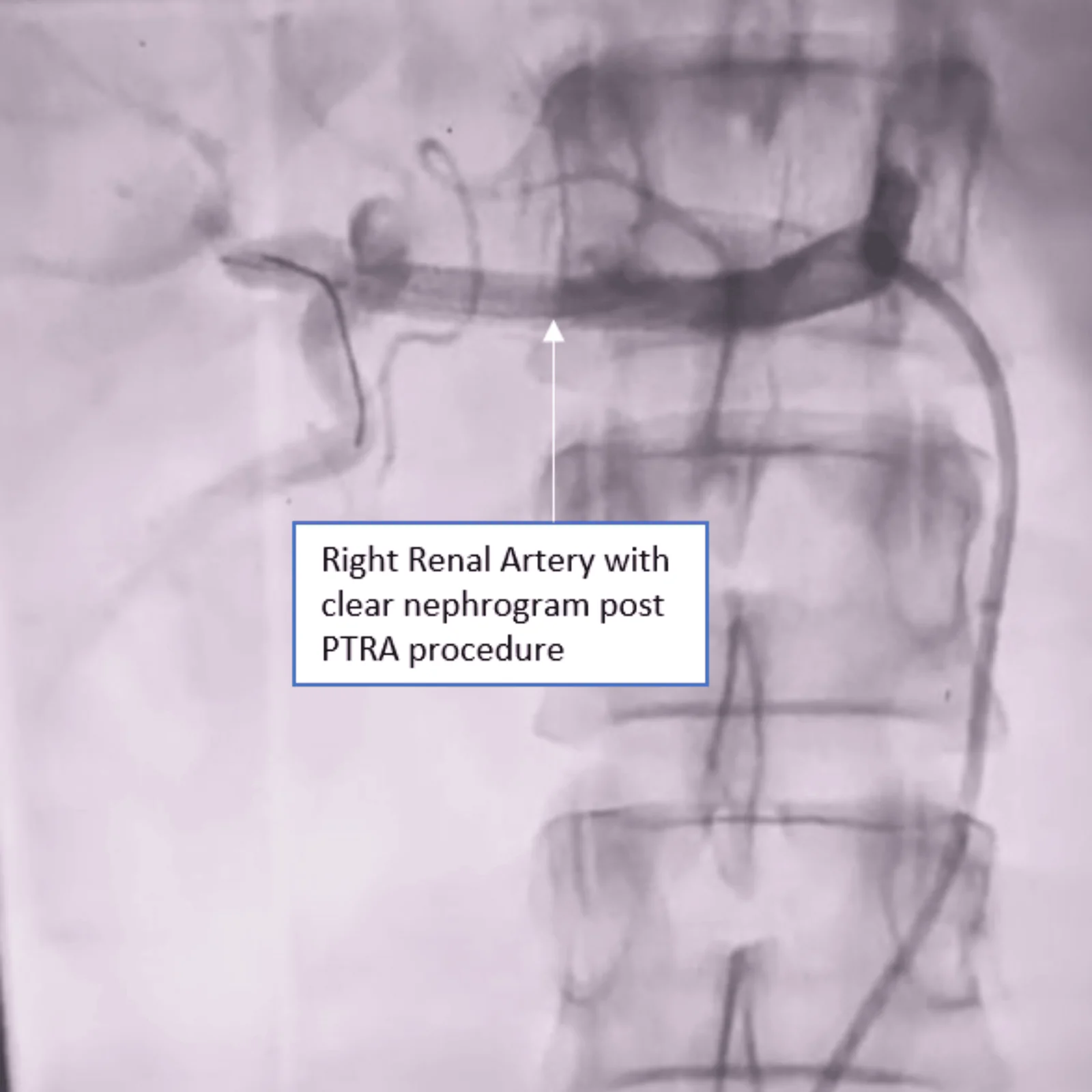
Right renal artery bloodflow restored after the percutaneous transluminal renal angioplasty (PTRA) procedure

Following the intervention, the patient required only two additional cycles of hemodialysis before demonstrating a significant improvement in urine output. He was successfully transitioned off dialysis, and his renal function tests completely normalized two months post-procedure.

## Discussion

This case illustrates the 'iceberg phenomenon of secondary hypertension', where critical vascular conditions remain occult until catastrophic renal failure occurs. Renal artery dissection combined with a pseudoaneurysm is an extremely rare and potentially lethal cause of AKI, as seen in this case.

Renal artery stenosis is a recognized and often underdiagnosed cause of secondary hypertension and progressive renal dysfunction. Atherosclerosis is the commonest cause of renal artery stenosis overall, while fibromuscular dysplasia is less common but is particularly relevant because it predisposes the patient to aneurysms and dissections [4]. The coexistence of stenosis, dissection, and pseudoaneurysm in a native renal artery is far less typical than isolated stenosis or transplant-related vascular complications [5].

A Doppler ultrasound is an appropriate first test, but a negative study does not exclude renal vasculopathy. CT angiogram or MR angiogram is generally the preferred second-line study when Doppler and the clinical picture disagree [6]. This case report makes this explicit, noting that the initial Doppler was unrevealing despite critical pathology and that repeat imaging only became diagnostic after clinical suspicion remained high.

A cross-sectional study reported a Doppler sensitivity of 79.4%, specificity of 84.6%, and overall accuracy of 81.7% using CT angiography as reference [7]. Ultrasound remains the first choice of investigation because of its noninvasive and inexpensive nature; however, its performance is operator-dependent and can be reduced by obesity, bowel gas, accessory vessels, distal lesions, or complex anatomy [8].

Renal artery pseudoaneurysms are rare (0.01%), while renal artery dissection accounts for 0.05% of all arterial dissections [9]. The incidence of simultaneous occurrence of all three pathologies in a single patient has not been reported to our knowledge.

In this patient, endovascular therapy (PTRA) was prioritized over open surgical options due to the critical progression of his acute kidney injury and the urgent need for reperfusion. Because the definitive pathology was delineated dynamically during the diagnostic angiogram in the catheterization lab, it afforded a critical window for immediate, same-setting intervention. Furthermore, PTRA provided a less invasive definitive treatment for an acutely ill patient, minimizing further procedural delays.

The patient presented with a family history of early-onset renal disease and long-term use of Rauwolfia serpentina; however, any mechanistic or epidemiologic link between these specific factors and the concurrent vascular pathology remains speculative. While chronic hypertensive injury and underlying genetic predisposition are theoretical contributors, a definitive causal association cannot be established in this case.

## Conclusions

Delayed recognition of renovascular pathology can lead to the burden of unnecessary long-term dialysis. In this instance, an initially unremarkable Doppler scan and an undisclosed family history obscured the diagnosis. This case serves as a vital reminder that in patients with a solitary functioning kidney and chronic hypertension, sudden anuria must trigger a high index of clinical suspicion. When initial non-invasive imaging is incongruent with severe clinical deterioration, physicians should promptly escalate to advanced cross-sectional imaging to rule out occult renovascular catastrophes.
